# Innominate Artery Graft Cannulation for Selective Antegrade Cerebral Perfusion in Aortic Surgery: Clinical Findings and Feasibility †

**DOI:** 10.3390/jcm14062126

**Published:** 2025-03-20

**Authors:** Ufuk Turkmen, Kudret Atakan Tekin, Gorkem Yigit, Ayla Ece Celikten, Ertekin Utku Unal

**Affiliations:** 1 Department of Cardiovascular Surgery, Faculty of Medicine, Hitit University, 19040 Corum, Turkey; gorkemyigit@hitit.edu.tr; 2 Department of Cardiovascular Surgery, Kosuyolu High Specialization Training and Research Hospital, 34865 Istanbul, Turkey; dr.kudretatakantekin@gmail.com; 3 Department of Cardiovascular Surgery, Erol Olcok Training and Research Hospital, 19040 Corum, Turkey; aecelikten@gmail.com; 4 Department of Cardiovascular Surgery, Dr. Ridvan Ege Hospital, Faculty of Medicine, Ufuk University, 06510 Ankara, Turkey; utkuunal@gmail.com

**Keywords:** aortic surgery, cerebral protection, innominate artery cannulation, Marfan syndrome, neurological complications, selective antegrade cerebral perfusion, Type A acute aortic dissection

## Abstract

**Background**: Cerebral protection during aortic surgery is crucial for improving surgical outcomes and reducing neurological complications. Selective antegrade cerebral perfusion (SACP) is increasingly used, and innominate artery (IA) side graft cannulation presents an innovative alternative to conventional axillary artery cannulation, with the potential to reduce complications associated with the latter. **Methods**: In this retrospective study, 196 patients who underwent proximal aortic surgery with IA graft cannulation for SACP between January 2021 and June 2024 were analyzed. Demographic data, intraoperative parameters, and postoperative outcomes were evaluated. Complications such as new stroke, postoperative delirium, mortality, and acute renal failure (ARF) were assessed. **Results**: The median age of the patients was 63 years, and 18.37% underwent emergency surgery for Type A acute aortic dissection (TAAAD). The most frequently performed surgical procedure was ascending aorta and hemiarch replacement (36.74%). The median cardiopulmonary bypass, cross-clamp, and SACP durations were 120.5, 93, and 23 min, respectively. The postoperative mortality rate was 3.06%, stroke rate was 2.04%, delirium rate was 9.18%, and ARF rate was 3.06%. All cases of delirium resolved spontaneously within 2–3 days. The mortality rate among Marfan syndrome (MFS) patients was 4.35%, with no reported stroke cases in this group. **Conclusions**: IA graft cannulation is a safe and effective method for providing SACP in aortic surgery, particularly in high-risk patient groups such as those with TAAAD and MFS. This technique ensures optimal cerebral perfusion, minimizes neurological and systemic complications, and enhances surgical efficiency by reducing operative duration. However, large-scale, multicenter, and prospective studies are needed to evaluate its long-term efficacy and safety.

## 1. Introduction

Ensuring cerebral protection during aortic surgery is crucial for improving surgical outcomes and reducing neurological complications. In procedures requiring circulatory arrest, the interruption of cerebral perfusion poses a significant risk for permanent neurological damage and mortality [1]. Therefore, selective antegrade cerebral perfusion (SACP) is increasingly being used to provide cerebral protection during circulatory arrest [2,3,4].

The arterial cannulation techniques used for SACP have evolved with technological advancements and clinical requirements. Traditionally, one of the most commonly used methods has been axillary artery (AX) cannulation, which has been considered an effective option for many years [5,6]. However, AX cannulation requires an infraclavicular incision and is associated with complications such as brachial plexus injury, seroma formation, and limb ischemia [7]. Additionally, in some cases, the limited diameter of the AX may be a restrictive factor, particularly in cases requiring total body perfusion.

To address these challenges, innominate artery (IA) cannulation has been increasingly preferred in recent years. This technique emerges as an alternative that can eliminate the need for additional incisions and associated complications required by AX cannulation [8]. IA cannulation is considered an effective option for ensuring the continuity of SACP while also providing a stable arterial inflow during cardiopulmonary bypass. The literature reports that IA cannulation offers advantages such as neuroprotection, maintenance of systemic perfusion, and ease of intraoperative management [9,10,11,12,13,14,15,16,17,18,19,20].

In this study, the efficacy and safety of SACP performed with IA graft cannulation were retrospectively evaluated. The impact of IA cannulation on neurological and systemic complications was analyzed and compared with other techniques reported in the literature. Our study aims to minimize surgical risks and enhance neurological outcomes by ensuring adequate and continuous cerebral perfusion through innominate artery graft cannulation, which allows for a stable and controlled antegrade blood flow during aortic surgery.

The preliminary results of this study were presented as an oral presentation at the 18th Turkish Society of Cardiovascular Surgery Congress (21–24 November 2024), and the abstract of the presentation was published in the official congress proceedings booklet [21]. However, the full text has not been previously published in any peer-reviewed journal. This manuscript significantly expands on the conference presentation by providing a more comprehensive analysis of intraoperative and postoperative outcomes.

## 2. Materials and Methods

### 2.1. Study Design and Patient Selection

This retrospective study included 196 patients who underwent proximal aortic surgery with SACP using IA graft cannulation between January 2021 and June 2024. The study was approved by the Ethics Committee of Hitit University Faculty of Medicine (Approval No: 2024-101) and was conducted in accordance with the principles of the Helsinki Declaration. Informed consent was obtained from all patients before treatment.

Patient data were retrospectively analyzed using electronic medical records and patient files. As a standard practice in our clinic, non-contrast thoracoabdominal computed tomography (CT) is performed preoperatively to assess for additional pathologies in elective patients scheduled for open-heart surgery. If any pathology is detected in any segment of the aorta, contrast-enhanced computed tomographic angiography (CTA) is performed to evaluate all major vascular structures and confirm the suitability of the IA for cannulation. In this study, all patients underwent preoperative CTA.

Coronary angiography (CAG) was performed in all elective patients over 40 years of age or with risk factors, except for those whose primary cardiac disease was coronary artery disease. However, patients with Type A acute aortic dissection (TAAAD) did not undergo CAG.

### 2.2. Study Definitions

Cross-clamp time: Defined as the duration from the application of the aortic cross-clamp to its removal.

SACP time: Defined as the duration from the application of the cross-clamp to the IA until its removal. The SACP time is a subset of the cross-clamp time.

New stroke: Defined as permanent brain injury that developed postoperatively and was confirmed by radiological imaging.

Postoperative delirium: Defined as a transient neurological condition characterized by fluctuations in consciousness, disorientation, agitation, confusion, restlessness, and uncontrolled behavior in the postoperative period.

Acute renal failure (ARF) refers specifically to patients who require hemodialysis in the postoperative period.

### 2.3. Inclusion and Exclusion Criteria (Figure 1)

#### 2.3.1. Inclusion Criteria:

- Patients who underwent aortic surgery with IA graft cannulation for SACP.
- Patients aged 20 years and older.
- Patients who underwent distal aortic anastomosis in an open fashion, were operated on at 28 °C, and underwent median sternotomy.

**Figure 1:**
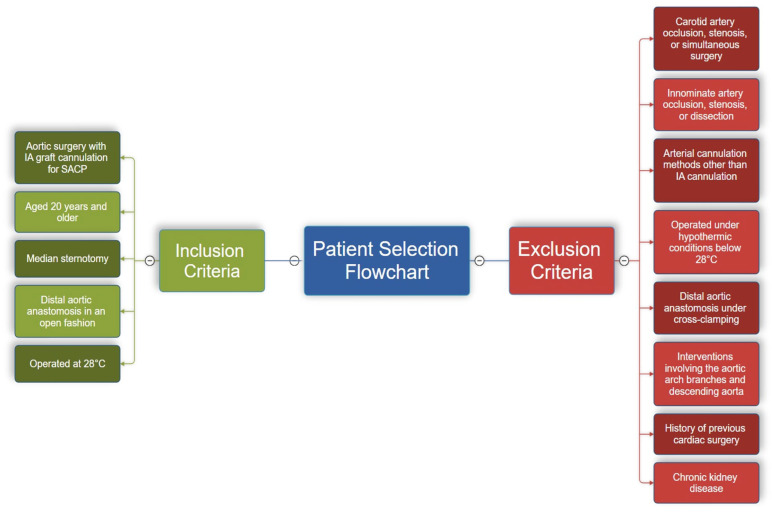
This flowchart illustrates the inclusion and exclusion criteria for patient selection in this study. Inclusion criteria are shown on the left in green, while exclusion criteria are displayed on the right in red. This structured selection process ensures a homogeneous study population, focusing on the safety and feasibility of IA cannulation in aortic surgery.

#### 2.3.2. Exclusion Criteria:

- Patients with occlusion or stenosis in the carotid arteries or those who underwent simultaneous carotid surgery.
- Patients with occlusion, stenosis, or dissection flap in the innominate artery.
- Patients who underwent arterial cannulation methods other than IA cannulation.
- Patients operated under hypothermic conditions below 28 °C.
- Patients who underwent distal aortic anastomosis under cross-clamping.
- Patients who underwent interventions involving the aortic arch branches and descending aorta.
- Patients with chronic kidney disease.
- Patients with a history of previous cardiac surgery.

These criteria were established to ensure the homogeneity of the study population and the consistency of the study’s scope.

### 2.4. Surgical Protocol and Technique

All patients underwent surgery under general anesthesia via median sternotomy. Central venous access was established with a central venous catheter inserted through the left jugular vein, and arterial blood pressure was monitored through the radial or brachial arteries.

To monitor cerebral oxygenation, non-invasive near-infrared spectroscopy (NIRS) was used with the O3 Regional Oximetry^®^ device (Masimo Corporation, Irvine, CA, USA).

**Figure 2:**
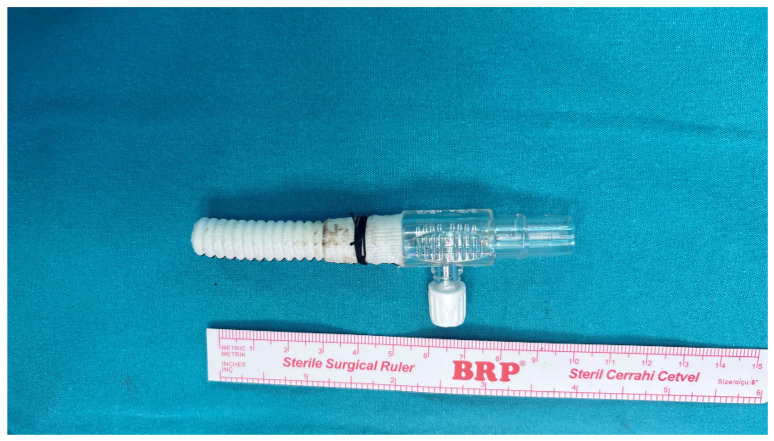
This image illustrates a 5 cm-long graft connected to a 3/8-inch connector, which is utilized for arterial cannulation in selective antegrade cerebral perfusion (SACP). The sterile surgical ruler displayed alongside the graft provides precise scaling for reference. The configuration demonstrates the compact and efficient design of the graft-connector assembly, emphasizing its practical application in achieving optimal blood flow during the procedure.

**Figure 3:**
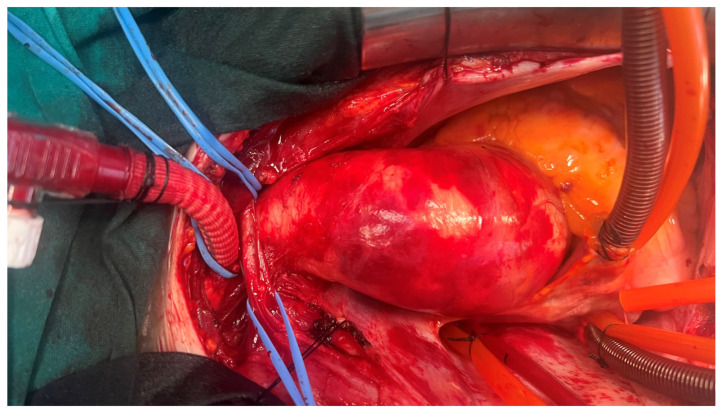
This image demonstrates an intraoperative view showing the successful anastomosis of a side graft to the innominate artery (IA). The graft is securely positioned within the surgical field, clearly illustrating its role in facilitating perfusion during the procedure.

The pericardium was opened, and the innominate vein (IV) was looped with two tapes. The entire aorta and its arch branches were explored, with only the IA being looped. A 5 cm long, 10 mm Dacron graft (Jotec FlowWeave BIOSEAL vascular graft^®^, Jotec GmbH, Hechingen, Germany) was attached to a 3/8-inch connector (Figure 2). After the administration of 300 IU/kg heparin, ensuring an activated clotting time (ACT) > 400 s, a partial side clamp was applied to the mid-segment of the IA. An incision was made on the IA, and the graft was anastomosed using a 6/0 polypropylene suture with a continuous technique reinforced with a Teflon felt. After removing the side clamp and ensuring air evacuation, the prepared graft was directly connected to the arterial return line via a 3/8-inch connector (Figure 3).

Venous return was achieved through either unicaval or bicaval cannulation. A left ventricular vent was inserted via the right superior pulmonary vein. Cardiopulmonary bypass (CPB) was initiated, and patients were cooled to 28 °C. Cross-clamping was performed, and cardiac arrest was induced using Del Nido cardioplegia and an ice slush. Cardioplegia was administered selectively through the antegrade route and/or directly into the coronary ostia. In patients with TAAAD or aortic valve insufficiency, additional retrograde cardioplegia was also used. In cases where the cross-clamp time exceeded 80 min, a repeat dose of Del Nido cardioplegia was administered at the 80th min.

After completion of the proximal aortic anastomosis and/or other surgical procedures, a cross-clamp was applied to the proximal IA before performing the distal aortic anastomosis. The patient was transitioned to SACP, and cerebral perfusion flow was maintained at 10–15 mL/kg/min. The left carotid and left subclavian arteries were clamped. The cross-clamp on the aorta was then removed, and the distal aortic anastomosis was performed in an open fashion.

Following the completion of the distal anastomosis, the cross-clamp on the IA was removed and repositioned onto the aortic graft. After weaning from CPB, the graft was ligated primarily and closed using a 3/0 polypropylene suture.

### 2.5. Statistical Analysis

Data were analyzed using IBM SPSS Statistics 23 software (IBM Inc., Chicago, IL, USA). Before initiating statistical analyses, data entry errors were checked, and all variables were assessed to ensure they were within the expected ranges.

Descriptive statistics were reported as mean ± standard deviation (Mean ± SD) for normally distributed continuous variables and as median (IQR: 25th–75th percentile) for non-normally distributed continuous variables. The assumption of normality was evaluated visually using histograms, Q-Q plots, skewness, and kurtosis values, and was statistically tested using the Shapiro–Wilk test.

Categorical variables were presented as frequencies (n) and percentages (%).

In this study, no statistical comparisons were made between the three patient groups (All Patients, TAAAD Group, and Marfan Syndrome Group). The aim of our study was to present the clinical and surgical outcomes of each group separately using descriptive statistics, rather than performing hypothesis testing. Therefore, statistical significance tests were not conducted, and *p*-values were not reported in the tables.

## 3. Results

### 3.1. Demographic Data

In this study, data from 196 patients were analyzed, with TAAAD and Marfan syndrome (MFS) patients evaluated as separate subgroups. The demographic and preoperative clinical characteristics of the patients are summarized in Table 1. A total of 36 patients (18.37%) underwent emergency surgery due to TAAAD. MFS was identified in 23 patients (11.73%). A total of 64 patients (32.65%) were female, with a median age of 63 years (IQR: 53–70). Only three patients (1.53%) had a history of cerebrovascular events (CVE), and the mean ejection fraction (EF) was calculated as 56.32 ± 7.37. Bicuspid aortic valve disease was detected in 44 patients (22.45%), with this rate rising to 73.91% among MFS patients. Hypertension was present in 61.2% of all patients, while it was observed in 91.7% of the TAAAD group.

### 3.2. Intraoperative Data

**Figure 4:**
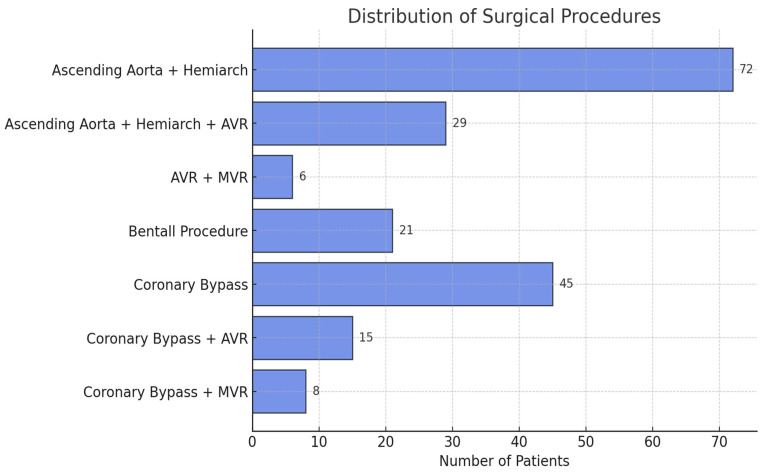
This bar chart illustrates the distribution of surgical procedures performed in the study population. Ascending aorta and hemiarch replacement was the most frequently performed procedure (72 patients), followed by coronary bypass surgery (45 patients). Other procedures included Bentall procedures, valve replacements (AVR, MVR), and combined procedures. This distribution reflects the diverse nature of surgical interventions in patients undergoing aortic surgery with IA cannulation.

The surgical procedures performed are presented in Table 2 and Figure 4. The most frequently performed surgical procedure was ascending aortic and hemiarch replacement in 72 patients (36.74%). Among MFS patients, the most common procedure was the Bentall operation, performed in 10 patients (43.48%). The median intraoperative times were recorded as follows: CPB time: 120.5 min (IQR: 81.75–154), cross-clamp time: 93 min (IQR: 61–131.25), and SACP time: 23 min (IQR: 20.75–24.25). The mean CPB flow rate was calculated as 4.54 ± 0.42 L/min.

### 3.3. Postoperative Data

The postoperative outcomes are presented in Table 3. No operative mortality was observed, while 30-day mortality was recorded in 6 patients (3.06%). Among these, three patients had undergone emergency surgery due to TAAAD. One patient with multiorgan failure died following hemiarch replacement, another patient could not be extubated due to a pulmonary infection, and a patient with a history of diplegia due to dissection who had undergone Bentall + hemiarch replacement was lost in the postoperative period.

Among the elective cases, the deceased patients included a patient with a history of cranial tumor surgery who did not regain consciousness postoperatively and died on postoperative day 2; a patient who underwent AVR (aortic valve replacement) and hemiarch replacement and subsequently developed mediastinitis; and a patient who underwent ascending aortic replacement and AVR but suffered a sudden cardiac arrest while under ward follow-up.

The median intubation duration was 8 h (IQR: 6–9) and the median intensive care unit (ICU) stay was 2 days (IQR: 2–3). Among postoperative complications, postoperative delirium was observed in 18 patients (9.18%), all of whom recovered spontaneously within 2–3 days. New-onset stroke was noted in four patients (2.04%), with complete recovery achieved in one case. Reoperation due to bleeding or tamponade was required in 12 patients (6.12%). ARF was detected in six patients (3.06%), four of whom had undergone emergency surgery for TAAAD. Postoperative atrial fibrillation (POAF) occurred in 45 patients (22.96%).

### 3.4. TAAAD Patient Group

Among the 36 patients who underwent surgery for TAAAD, the median age was calculated as 57 years (IQR: 52.25–69.25). The median CPB duration was 83.5 min (IQR: 80.75–118.5) and the median SACP duration was 23 min (IQR: 21–25.25). The most frequently performed surgical procedure was ascending aortic and hemiarch replacement in 28 patients (77.77%). Postoperative mortality was observed in three patients (8.33%), stroke in one patient (2.78%), and ARF in four patients (11.11%).

### 3.5. Marfan Syndrome Group

Among the 23 patients with MFS, the median age was 45 years (IQR: 38–52). The median cross-clamp duration was 104 min (IQR: 65–135). The most frequently performed surgical procedure was the Bentall operation, conducted in 10 patients (43.48%). Mortality was recorded in one patient (4.35%), and no stroke was observed in this group. ARF occurred in two patients (8.7%), while postoperative delirium was noted in three patients (13.04%).

### 3.6. Overall Assessment

In this study, TAAAD and MFS patients were analyzed as separate subgroups without direct comparison. While TAAAD patients exhibited higher mortality and complication rates due to the emergency nature of the surgery, postoperative outcomes in MFS patients were more favorable despite longer surgical durations.

## 4. Discussion

In our study, we found that innominate artery (IA) graft cannulation using a 10 mm side graft is a safe and feasible technique for aortic surgery. This approach ensured stable and adequate cerebral perfusion, effectively supporting both systemic and neurological outcomes. The observed rates of stroke, postoperative delirium, and acute kidney injury were comparable to or lower than those reported in previous studies, further supporting the efficacy of IA cannulation as an alternative perfusion strategy. Additionally, our findings suggest that this technique may be particularly beneficial in high-risk patient groups, such as those with acute Type A aortic dissection (TAAAD) and Marfan syndrome (MFS).

The introduction of SACP via AX cannulation has marked a significant advancement in aortic surgery [5]. Traditionally, AX cannulation has been considered the standard method for SACP. However, this technique is associated with complications such as brachial plexus injury, seroma formation, and limb ischemia [5,7]. Additionally, the smaller diameter of the AX can lead to high pressure in the arterial return line, prolonging the cooling and rewarming phases, which may contribute to increased operative times [6].

It has been reported that IA and AX cannulation provide comparable levels of neuroprotection and can be used interchangeably depending on surgical conditions [9]. However, some studies suggest that IA cannulation is equivalent to or even superior to AX cannulation in certain cases [10]. Direct IA cannulation is a widely used technique for both CPB and SACP. While it offers the advantage of rapid application, the manipulation of large-diameter cannulas carries the risk of vascular injury and cerebral embolism. To mitigate this risk, studies using smaller cannulas (7–14 Fr) have reported successful outcomes. However, the use of smaller cannulas may necessitate additional cannulation for systemic circulation [15,16,18,19].

The side graft technique eliminates cannula manipulation, reducing the risk of vascular injury and cerebral embolism while effectively maintaining both systemic and cerebral circulation. In this technique, 22 or 24 Fr arterial cannulas are inserted into the side graft [11,13,14], while some studies have connected the side graft directly to the arterial return line [9,10,12,18,20]. Nevertheless, some studies indicate no significant differences in neurological complications, mortality, or morbidity between direct IA cannulation and the side graft technique [17,18].

The use of a 10 mm side graft for IA cannulation has provided significant advantages in both the operative process and the postoperative period in our study. Connecting the 10 mm graft to the arterial return line via a 3/8-inch connector offers optimal blood flow, presenting a crucial advantage. The narrowest part of the arterial return line, the connector, has an inner diameter of 9.52 mm, while a 24Fr arterial cannula has an inner diameter of 8 mm, resulting in a 19% wider flow area. This advantage of the larger-diameter graft can accelerate the cooling and rewarming processes while simultaneously optimizing both systemic and cerebral circulation. In our study, the median CPB time was recorded as 120.50 min (IQR: 81.75–154), the cross-clamp time as 93 min (IQR: 61–131.25), and the SACP time as 23 min (IQR: 20.75–24.25) (Table 2). These recorded durations suggest that this technique has the potential to reduce surgical trauma and minimize complications that may arise during circulatory arrest. Even in patients with the highest BSA (2.43 m^2^) and cardiac output (5.83 L/min) in our cohort, CPB was maintained smoothly with minimal pressure in the arterial outflow line, highlighting the significant advantage of IA graft cannulation.

In addition to these positive effects, the postoperative 30-day mortality rate in our study was determined to be 3.06% (six patients). Among these patients, three underwent emergency surgery due to TAAAD; one died from multiorgan failure, another from pulmonary complications, and the third from hypovolemic shock. Among elective cases, three patients succumbed to postoperative complications: one due to mediastinitis and the other two due to severe cardiac complications. In the literature, similar studies including both elective and emergency cases with IA side graft cannulation report mortality rates ranging from 2.30% to 7.10%. Mert et al. reported a mortality rate of 7.10% (5 deaths in 70 patients), Preventza et al. reported 4.90% (13 deaths in 263 patients), Eldeiry et al. reported 2.30% (3 deaths in 129 patients), Ünal et al. reported 6.25% (2 deaths in 32 patients), and Di Eusanio et al. reported 3.60% (2 deaths in 55 patients) [10,11,12,13,14]. The mortality rate of 3.06% (6 deaths in 196 patients) observed in our study is at the lower end of this range, supporting the safety and efficacy of IA cannulation as a viable surgical technique.

ARF was observed in a total of six patients (3.06%), including four who underwent emergency surgery. All of these patients experienced postoperative hemodynamic instability and required temporary hemodialysis. In the literature, similar studies have reported ARF rates ranging from 0.80% to 15.63%. Mert et al. reported an ARF rate of 4.2% (3 cases in 32 patients), Preventza et al. reported 9.10% (24 cases in 263 patients), Eldeiry et al. reported 0.80% (1 case in 129 patients), Ünal et al. reported 15.63% (5 cases in 32 patients), and Di Eusanio et al. reported 1.80% (1 case in 55 patients) [10,11,12,13,14]. The ARF rate of 3.06% (6 cases) observed in our study is within this range and supports the potential of IA cannulation in reducing the risk of postoperative renal injury.

Although we routinely used NIRS, regional cerebral oxygen saturation (rSO_2_) values were not recorded in most cases. However, we observed that when partial cross-clamping was applied to the IA, the right rSO_2_ values, or both right and left rSO_2_ values during SACP, did not decrease by more than 20%, as reported by Slater et al. [22].

In the postoperative period, stroke was observed in a total of four patients (2.04%), with one patient showing full neurological recovery in response to treatment. In the literature, stroke rates in similar studies have been reported to range from 0% to 3.4%. Mert et al. reported a stroke rate of 1.4% (1 case in 70 patients), Preventza et al. reported 3.4% (9 cases in 263 patients), Eldeiry et al. reported 2.3% (3 cases in 129 patients), while both Di Eusanio et al. (55 patients) and Ünal et al. (32 patients) reported a 0% stroke rate [10,11,12,13,14]. The stroke rate of 2.04% observed in our study falls within this range and remains at a low level. The effectiveness of cerebral perfusion protocols during IA cannulation may be a significant factor contributing to the low stroke rate in our study. A meticulous perfusion strategy ensuring adequate oxygen delivery to both hemispheres during SACP may have played a key role in reducing neurological complications. Additionally, the direct arterial flow provided by IA cannulation and the maintenance of stable cerebral perfusion suggests that this technique may be an effective approach for neurological protection.

Additionally, postoperative delirium was observed in 18 patients (9.18%), and all cases resolved spontaneously within 2–3 days. In our study, postoperative delirium was defined as a broad clinical spectrum, including postoperative confusion, agitation, altered consciousness, disorientation, restlessness, and uncontrollable behavior. Therefore, it should be considered that some studies in the literature may have used narrower definitions. Reported rates of postoperative delirium in similar studies range from 1.4% to 7.8%. Preventza et al. reported a rate of 3.8% (10 cases in 263 patients), Eldeiry et al. 7.8% (10 cases in 129 patients), Mert et al. 1.4% (1 case in 70 patients), Di Eusanio et al. 1.8% (1 case in 55 patients), and Ünal et al. 6.25% (2 cases in 32 patients) [10,11,12,13,14]. The postoperative delirium rate of 9.18% in our study is at the upper limit of the values reported in the literature. However, considering the broad definition used in our study and the fact that all delirium cases were resolved within a short period, this supports the hypothesis that IA cannulation is an effective method for cerebral protection and has the potential to reduce postoperative neurological morbidity.

There is limited information in the literature regarding the use of IA cannulation in aortic surgery for patients with connective tissue disorders such as Marfan syndrome (MFS) [15,16,17,20]. In our study, MFS was identified in 23 patients (11.73%), six of whom underwent emergency surgery due to TAAAD. The median cross-clamp time was 104 min (IQR: 65–135), which may be associated with the prolonged surgical duration of the Bentall procedure. The 30-day mortality rate in MFS patients was 4.35%, observed in a patient who underwent emergency surgery for TAAAD. Neurological complications were limited to postoperative delirium in three patients (13.04%), with no other neurological issues reported in this group. Additionally, ARF was detected in two patients (8.7%). IA graft cannulation was successfully performed in all patients whose preoperative CTA confirmed the absence of IA pathology. Our study findings support IA cannulation as a safe and effective option for MFS patients. If confirmed by larger-scale studies, IA cannulation may be considered a standard approach in this patient population.

Early mortality rates in patients undergoing TAAAD repair range from 17% to 26% [23]. This high mortality rate necessitates careful selection of the arterial cannulation technique. The correct cannulation site is critical for ensuring adequate blood flow to the true lumen and minimizing the risk of malperfusion. The feasibility of IA cannulation in TAAAD is primarily determined by the absence of dissection, atherosclerosis, or stenosis in the IA on preoperative imaging, making this assessment a key factor in decision-making.

Studies on direct IA cannulation have demonstrated its safety and efficacy. In the series by Kong et al., which included 29 patients, the reported mortality rate was 3.40%, the stroke rate was 6.90%, the ARF rate was 0%, and the confusion rate was 13.8% [15]. Payabyab et al. reported a mortality rate of 14.70%, a stroke rate of 9.30%, and an ARF rate of 14.70% in their study involving 75 patients [16]. Feier et al., in a series of 34 patients, documented a mortality rate of 8.82%, a stroke rate of 3.12%, and an ARF rate of 3.12% [17]. Additionally, in a study by Eldeiry et al. including 22 patients who underwent IA cannulation with a side graft, the mortality rate was 9.10%, the stroke rate was 9.10%, the confusion rate was 13.6%, and the ARF rate was 0% [10].

The outcomes observed in our study for the TAAAD group undergoing IA graft cannulation are largely consistent with these findings. Among the 36 patients who underwent surgery for TAAAD, the postoperative 30-day mortality rate was 8.33% (3 patients), the stroke rate was 2.78% (1 patient), the postoperative delirium rate was 16.67% (6 patients), and the ARF rate was 11.11% (4 patients).

The comparative analyses of IA and AX cannulation techniques further support the reliability of IA cannulation. In the study by Eldeiry et al., no significant difference was found between IA and AX cannulation in emergency cases [10]. Payabyap et al. reported that in their study of 75 patients undergoing IA cannulation, dissection extended into the IA in seven cases, but this did not pose an obstacle to cannulation [16]. Feier et al. emphasized that IA cannulation provided better perfusion, reduced postoperative complications, and decreased mortality in TAAAD patients [17]. Kong et al. stated that direct IA cannulation is a simple, reliable, and effective method for providing SACP during total arch replacement and frozen elephant trunk techniques [15].

In conclusion, the findings of our study demonstrate that IA cannulation with a side graft is a safe and effective technique in TAAAD surgery. Compared to previously reported studies on IA cannulation, this technique appears to offer advantages in terms of lower rates of neurological and renal complications, as well as reduced mortality. The results of our study support the feasibility and reliability of IA cannulation as a viable option for TAAAD patients.

One of the key advantages of IA cannulation is the continuous perfusion to the right arm during surgery. Ischemic complications associated with AX cannulation have been reported in the literature [5,6,7], but IA cannulation reduces this risk, offering a safer alternative. Additionally, securing hemostasis by ligating the graft after CPB termination simplifies bleeding control and may shorten the overall operative time. Furthermore, the simplicity of the cannulation procedure facilitates the decannulation process, reducing sternum closure time and accelerating patient rewarming.

In the literature, AX cannulation has been associated with a higher postoperative blood transfusion requirement, which may contribute to coagulopathy [10]. Although our study did not specifically record the volume of postoperative blood transfusion, the relatively short graft length (~5 cm) may help reduce intraoperative blood loss and maintain hemodynamic stability. Moreover, keeping the graft within the surgeon’s visual field minimizes potential complications such as intraoperative graft compression and mobilization, making the cannulation process safer and more feasible. However, further studies are needed to determine the precise impact of IA cannulation on postoperative blood transfusion requirements.

During SACP with AX cannulation, the IA and arch branches need to be mobilized for cross-clamping. Therefore, performing a side graft anastomosis to an already mobilized healthy IA is a more practical and effective alternative than creating an additional axillary incision. These advantages highlight the crucial role of IA cannulation in enhancing surgical safety and efficacy.

## 5. Limitations

This study has several important limitations. First, due to its retrospective design, data collection may have been subject to inconsistencies and biases. Specifically, intraoperative regional cerebral oxygen saturation (rSO_2_) values were not recorded for most cases, limiting the objective assessment of the impact of IA cannulation on cerebral oxygenation.

The lack of specific records on postoperative blood transfusion volumes prevents a clear analysis of the effects of IA cannulation on bleeding control and transfusion requirements. Although previous studies have reported that AX cannulation may increase the risk of coagulopathy and bleeding, a direct comparison could not be made in our study.

Since our study was conducted at a single center, the generalizability of the results is limited. The efficacy and safety of IA cannulation should be evaluated in future studies involving different surgical centers and patient populations. Moreover, although we analyzed the outcomes of patients who underwent IA graft cannulation, a direct comparison with alternative techniques such as AX cannulation or direct IA cannulation was not performed. This limitation makes it difficult to draw definitive conclusions regarding the superiority or disadvantages of IA cannulation relative to other techniques.

The definition of postoperative delirium in our study encompassed a broad clinical spectrum. In the literature, some studies have used narrower definitions, which may complicate direct comparisons of incidence rates across studies.

In terms of patient selection, the study included heterogeneous patient groups, and TAAAD and MFS patients were not directly compared. Particularly, the long-term effects of IA cannulation in connective tissue disorders such as MFS require further investigation in more comprehensive studies.

Finally, this study primarily focused on early postoperative outcomes. To obtain more robust data on the long-term efficacy and safety of IA cannulation, large-scale, multicenter, and prospective studies are warranted.

## 6. Conclusions

This study demonstrates that innominate artery (IA) cannulation using a 10 mm side graft is a safe and feasible technique in aortic surgery. This approach ensures optimal and stable perfusion, effectively supporting both cerebral and systemic circulation while minimizing the risk of neurological complications.

The observed favorable outcomes in high-risk patient groups, including acute Type A aortic dissection (TAAAD) and Marfan syndrome (MFS), support the safe application of this technique. However, as this study has a retrospective design, further large-scale, multicenter, and prospective studies are required to provide stronger evidence regarding the long-term efficacy and safety of IA cannulation.

## Figures and Tables

**Table 1:** Demographic and clinical characteristics of patients.

Variables	All Patients (n = 196)	TAAAD Group (n = 36)	Marfan Syndrome Group (n = 23)
Female sex	64 (32.65%)	13 (36.11%)	4 (17.39%)
Age (years)	63 (IQR: 53–70)	57 (IQR: 52–69)	45 (IQR: 38–52)
BSA (kg/m^2^)	1.89 ± 0.17	1.93 ± 0.15	1.93 ± 0.14
BMI	29.4 (IQR: 26.3–33.2)	28.5 (IQR: 26.2–31.2)	26.7 (IQR: 24.6–29.5)
EF %	56.32 ± 7.37	53.6 ± 5.0	53.7± 8.1
Diabetes Mellitus	70 (35.71%)	9 (25%)	6 (26.09%)
TAAAD	36 (18.37%)	36 (100%)	6 (26.09%)
Hypertension	120 (61.22%)	33 (91.7%)	10 (43.48%)
COPD	37 (18.8%)	7 (19.4%)	1 (4.35%)
History of CVE	3 (1.53%)	0 (0%)	0 (0%)
Bicuspid Aortic Valve	44 (22.45%)	7 (19.4%)	17 (73.91%)
Aortic Valve Disease			
None	125 (63.77%)	29 (80.6%)	6 (26.08%)
Stenosis	32 (16.33%)	3 (8.3%)	1 (4.35%)
Regurgitation	39 (19.90%)	4 (11.1%)	16 (69.57%)
Marfan Syndrome	23 (11.73%)	6 (16.67%)	23 (100%)

BSA: body surface area; BMI: body mass index; EF: ejection fraction; IQR: 25th–75th percentile; TAAAD: Type A Acute Aortic Dissection; COPD: chronic obstructive pulmonary disease; CVE: cerebrovascular events.

**Table 2:** Intraoperative data.

Variables	All Patients (n = 196)	TAAAD Group (n = 36)	Marfan Syndrome Group (n = 23)
Type of Surgery (Additional Procedure)			
Ascending aorta and hemiarch replacement	72 (36.74%)	28 (77.77%)	6 (26.08%)
Ascending aorta and hemiarch replacement + AVR	29 (14.80%)	3 (8.33%)	7 (30.44%)
AVR + MVR	6 (3.06%)	0 (0%)	0 (0%)
Bentall Procedure	21 (10.71%)	3 (8.33%)	10 (43.48%)
Coronary bypass	45 (22.96%)	1 (2.78%)	0 (0%)
Coronary bypass + AVR	15 (7.65%)	1 (2.78%)	0 (0%)
Coronary bypass + MVR	8 (4.08%)	0 (0%)	0 (0%)
Cardiopulmonary Bypass Time (min)	120.50 (IQR: 81.75–154)	83.5 (IQR: 80.75–118.5)	127 (IQR: 86–150)
Cross-clamp Time (min)	93 (IQR: 61–131.25)	64 (IQR: 59.75–79)	104 (IQR: 65–135)
SACP Time (minutes)	23 (IQR: 20.75–24.25)	23 (IQR: 21–25.25)	22 (IQR: 19.5–25)
Perfusion Flow Rate (L/min)	4.54 ± 0.42	4.62 ± 0.36	4.63 ± 0.33

SACP: Selective Antegrade Cerebral Perfusion; AVR: Aortic Valve Replacement; MVR: Mitral Valve Replacement; IQR: 25th–75th percentile.

**Table 3:** Postoperative data.

Variables	All Patients (n = 196)	TAAAD Group (n = 36)	Marfan Syndrome Group (n = 23)
30-day Mortality	6 (3.06%)	3 (8.33%)	1 (4.35%)
Reoperation	12 (6.12%)	4 (11.11%)	3 (13.04%)
Stroke	4 (2.04%)	1 (2.78%)	0 (0%)
Postoperative Delirium	18 (9.18%)	6 (16.67%)	3 (13.04%)
Acute Renal Failure	6 (3.06%)	4 (11.11%)	2 (8.7%)
Sternal Infection	2 (1.02%)	0 (0%)	0 (0%)
POAF	45 (22.96%)	10 (27.78%)	5 (21.26%)
Intubation Duration (h)	8 (IQR: 6–9)	9.5 (IQR: 9–12)	15 (IQR: 6–30)
ICU Stay (days)	2 (IQR: 2–3)	3 (IQR: 3–3)	2.7 (IQR: 2–3)
Length of Stay in Postoperative Ward (days)	9 (IQR: 8–9)	10 (IQR: 9–10)	8.4 (IQR: 8–10)
Total Hospital Stay (days)	11 (IQR: 10–12)	13 (IQR: 12–13)	11.1 (IQR: 10–12)

POAF: Postoperative Atrial Fibrillation; ICU: Intensive Care Unit; IQR: 25th–75th percentile.

## Data Availability

The data that support the findings of this study are not publicly available due to ethical and privacy restrictions. However, they can be obtained from the corresponding author upon reasonable request and with the approval of the institutional ethics committee.

## Abbreviations

The following abbreviations are used in this manuscript:

ACT	Activated clotting time
ARF	Acute renal failure
AX	Axillary artery
AVR	Aortic valve replacement
BMI	Body mass index
BSA	Body surface area
CAG	Coronary angiography
CPB	Cardiopulmonary bypass
COPD	Chronic obstructive pulmonary disease
CTA	Computed tomographic angiography
CT	Computed tomography
CVE	Cerebrovascular events
EF	Ejection fraction
IA	Innominate artery
ICU	Intensive care unit
IQR	Interquartile range (25th–75th percentile)
IV	Innominate vein
MFS	Marfan syndrome
MVR	Mitral valve replacement
NIRS	Non-invasive near-infrared spectroscopy
POAF	Postoperative atrial fibrillation
SACP	Selective antegrade cerebral perfusion
TAAAD	Type A acute aortic dissection
